# Mutations in cell elongation genes *mreB, mrdA* and *mrdB* suppress the shape defect of RodZ-deficient cells

**DOI:** 10.1111/mmi.12148

**Published:** 2013-01-21

**Authors:** Daisuke Shiomi, Atsushi Toyoda, Tomoyuki Aizu, Fumio Ejima, Asao Fujiyama, Tadasu Shini, Yuji Kohara, Hironori Niki

**Affiliations:** 1 Microbial Genetics Laboratory, Genetic Strains Research Center, National Institute of Genetics1111 Yata, Mishima, Shizuoka, 411-8540, Japan; 2 Comparative Genomics Laboratory, National Institute of Genetics1111 Yata, Mishima, Shizuoka, 411-8540, Japan; 3 Genetic Biology Laboratory, National Institute of Genetics1111 Yata, Mishima, Shizuoka, 411-8540, Japan; 4 Department of Genetics, The Graduate University for Advanced Studies, Sokendai1111 Yata, Mishima, Shizuoka, 411-8540, Japan

## Abstract

RodZ interacts with MreB and both factors are required to maintain the rod shape of *Escherichia coli*. The assembly of MreB into filaments regulates the subcellular arrangement of a group of enzymes that synthesizes the peptidoglycan (PG) layer. However, it is still unknown how polymerization of MreB determines the rod shape of bacterial cells. Regulatory factor(s) are likely to be involved in controlling the function and dynamics of MreB. We isolated suppressor mutations to partially recover the rod shape in *rodZ* deletion mutants and found that some of the suppressor mutations occurred in *mreB*. All of the *mreB* mutations were in or in the vicinity of domain IA of MreB. Those *mreB* mutations changed the property of MreB filaments *in vivo*. In addition, suppressor mutations were found in the periplasmic regions in PBP2 and RodA, encoded by *mrdA* and *mrdB* genes. Similar to MreB and RodZ, PBP2 and RodA are pivotal to the cell wall elongation process. Thus, we found that mutations in domain IA of MreB and in the periplasmic domain of PBP2 and RodA can restore growth and rod shape to Δ*rodZ* cells, possibly by changing the requirements of MreB in the process.

## Introduction

In the 1970s, there were many attempts to identify cytoskeletal proteins in prokaryotes (Minkoff and Damadian, 1976; Beck *et al*., 1978; Nakamura and Watanabe, 1978; Nakamura *et al*., 1978). However, none had shown conclusive evidence that *Escherichia coli* possesses cytoskeletal proteins. A breakthrough that led to the discovery of bacterial cytoskeletal proteins came from studies of cell division and morphology, and now the gene products of *ftsZ* and *mreB* in prokaryotes are known to be structurally and functionally related to eukaryotic tubulin and actin respectively (Wachi *et al*., 1987; Bi and Lutkenhaus, 1991; de Boer *et al*., 1992; RayChaudhuri and Park, 1992; Lowe and Amos, 1998; Jones *et al*., 2001; van den Ent *et al*., 2001). FtsZ is a bacterial tubulin, an essential protein that assembles first at a division site to form a ring-like structure (Z ring) that recruits multiple proteins involved in cell division such as ZipA and FtsA (Adams and Errington, 2009). Cells lacking functional FtsZ become filamentous because they are not able to divide. On the other hand, a mutation in *mreB* has been found that causes a defect in cell morphology (Wachi *et al*., 1987; Jones *et al*., 2001). MreB proteins assemble to form filamentous polymers or patches on the periphery of the cell membrane (Jones *et al*., 2001; Kruse *et al*., 2003; Shih *et al*., 2003). Furthermore, purified MreB from *Thermotoga maritima* assembles into double filaments on a membrane surface (Salje *et al*., 2011). It has been shown recently that MreB rotates around the long axis of the cell, and that the rotation is dependent on the assembly of the cell wall but not on assembly of MreB filaments (Dominguez-Escobar *et al*., 2011; Garner *et al*., 2011; van Teeffelen *et al*., 2011). In addition to FtsZ and MreB, it has been shown that bacterial cells have other cytoskeletal proteins that are intermediate filament-like proteins (Ausmees *et al*., 2003).

The cytoskeletal proteins FtsZ and MreB are essential for maintenance of proper cell shape. In addition, RodZ that genetically interacts with FtsZ and physically interacts with MreB has been found recently (Shiomi *et al*., 2008; Alyahya *et al*., 2009; Bendezu *et al*., 2009). A deletion mutant of the *rodZ* results in cells becoming round or misshapen. In contrast, overproduction of RodZ results in elongation of the cell. RodZ shows spotty patterns along the long axis of the cell and colocalizes with MreB (Shiomi *et al*., 2008; Alyahya *et al*., 2009; Bendezu *et al*., 2009). A co-crystal structure of MreB and the cytoplasmic domain of RodZ from *T. maritima* has been solved (van den Ent *et al*., 2010). RodZ has a helix–turn–helix (HTH) motif at the N-terminus that is required for interaction with MreB. It is still unclear whether RodZ is required for assembly or proper localization of MreB (Shiomi *et al*., 2008; Alyahya *et al*., 2009; Bendezu *et al*., 2009) Regardless, RodZ is deeply involved in the regulation of cell length and width to maintain the rod shape through regulation of peptidoglycan synthesis of the central cylinder of the rod (Shiomi *et al*., 2008; 2009).

Peptidoglycan is a giant macromolecule consisting of glycan strands cross-linked by short peptides. It surrounds cells and protects them against various stresses such as osmotic pressure (Vollmer and Bertsche, 2008; Vollmer *et al*., 2008a,b). It is synthesized by multiple enzymes, including penicillin binding proteins (PBPs) (Popham and Young, 2003). Purified sacculi from *E. coli* still retain their rod shape (de Pedro *et al*., 1997), suggesting that the shape of peptidoglycan determines the shape of the cell. Therefore, it is very important for a cell to regulate the activities and localization of PBPs to synthesize peptidoglycan correctly. PBP2, encoded by *mrdA*, is a specific enzyme that synthesizes the peptidoglycan of the cylinder (Spratt, 1975) and functions with RodA, encoded by *mrdB* (Ishino *et al*., 1986). PBP2 is a bitopic membrane protein, while RodA is a polytopic membrane protein. PBP2 and RodA show spotty patterns along the long axis of the cell (Den Blaauwen *et al*., 2003; Vats *et al*., 2009). Peptidoglycan synthesis is regulated by a complex containing MreB, PBP2 and RodA during cell elongation.

MreB consists of two major domains (I and II) and each domain is further divided into two subdomains (IA, IB, IIA and IIB) (van den Ent *et al*., 2001). This domain organization is very similar to eukaryotic actin. It seems that the top regions of IB and IIB domains interact with the bottom regions of domain IA and IIA to form MreB filaments (van den Ent *et al*., 2001; Oda *et al*., 2009). It is thought that domain IA of actin as well as bacterial actin play an important role in filament assembly. New peptidoglycan of the cylinder is actively synthesized along the MreB polymers in *Bacillus subtilis* (Daniel and Errington, 2003). It seems likely that the internal cytoskeletal filament of MreB can govern the distribution of periplasmic enzymes for peptidoglycan synthesis. It is likely that the transmembrane proteins RodZ and MreC connect them because bacterial two-hybrid assays indicate that there are interactions among RodZ, MreB, MreC and PBP2 (Kruse *et al*., 2005; Bendezu *et al*., 2009). The cytoplasmic region of RodZ can interact with MreB and the periplasmic region can affect the rod shape, suggesting an interaction of the enzymes for peptidoglycan synthesis. It is thought that RodZ could act as an intermediate between MreB and the enzymes for peptidoglycan synthesis to elongate peptidoglycan.

To understand how RodZ determines the cell shape, we isolated suppressor mutants of *rodZ* mutant cells whose growth was faster and had a restored cell shape in rich medium. We sequenced the whole genomes of the suppressor strains to map mutation sites by the next-generation Solexa sequencer. Most of them were mapped to *mreB*, *mrdA* or *mrdB*. All of these proteins have been shown to be involved in the synthesis of cylindrical peptidoglycan (Spratt, 1975; Ishino *et al*., 1986; Uehara and Park, 2008), which is consistent with the idea that RodZ is involved in its synthesis. Interestingly, all of the *mreB* mutations were found in or proximal to domain IA of MreB. We also discuss the function of domain IA of MreB and the significance of all the mutations of PBP2 and RodA being found in the periplasmic domain.

## Results

### Isolation of mutants to suppress the slow-growth phenotype of the *rodZ* mutant

**Fig. 1:**
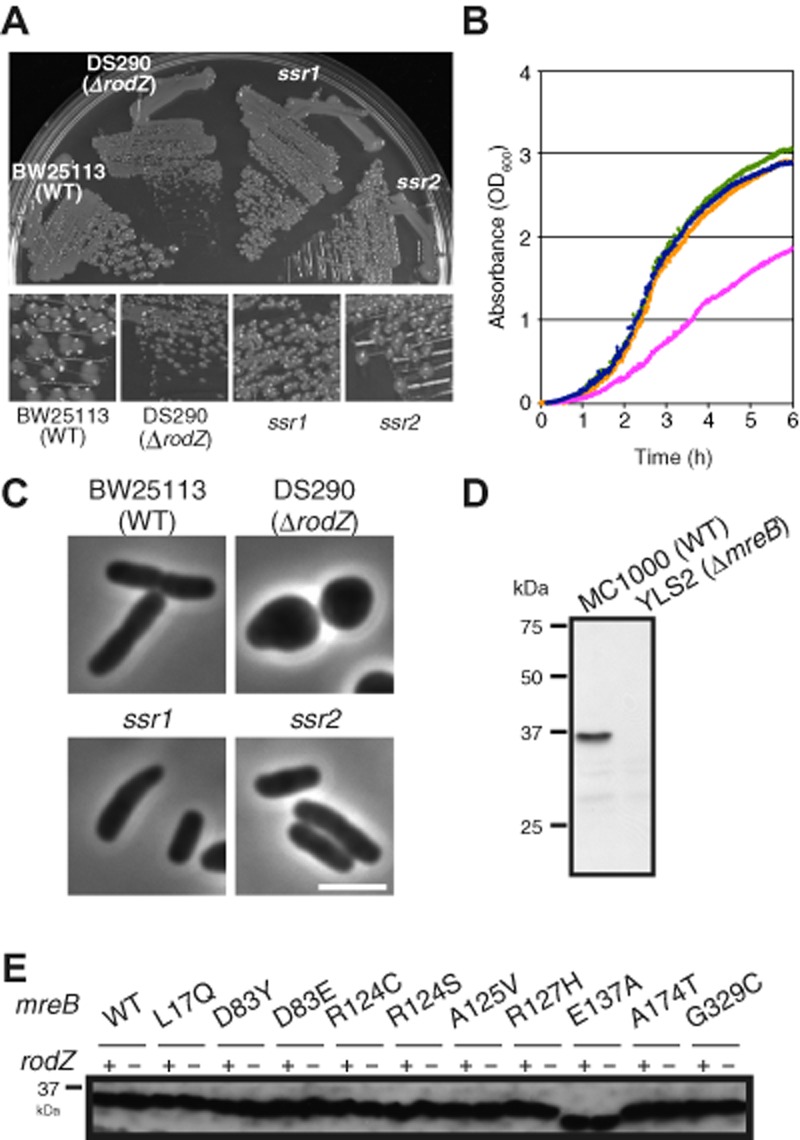
Growth of the wild type, *rodZ* mutant and suppressors of slow-growth phenotype of the *rodZ* mutant, and expression of MreB in the *ssr* mutants. A. Cells were streaked on L plates and the plate was incubated at 37°C for 12 h. Magnified images are shown below. B. Cell growth of BW25113 (WT) (blue), DS290 (Δ*rodZ*) (magenta), ssr1 (orange) and ssr2 (green) in L broth at 37°C. Absorbance (OD_600_) was recorded automatically by a Bio-photorecorder (TVS062CA, Advantech). C. Phase contrast images of BW25113, DS290, ssr1 and ssr2 grown in L broth at 37°C to log phase. D. Immunoblot of MC1000 (wild type) and YLS2 (MC1000 Δ*mreB*) using anti-MreB antiserum. YLS2 was kindly provided by Dr Rothfield (University of Connecticut). E. Immunoblots of *rodZ*^+^ or Δ*rodZ* cells producing each MreB mutant using anti-MreB antiserum.

Growth of the *rodZ* mutant strain is significantly slower than that of the wild-type strain (BW25113) (Shiomi *et al*., 2008). Thus, it is possible to select a suppressor mutant that can grow faster than the original *rodZ* mutant strain. To isolate suppressor mutants for the growth defect, single colonies of *rodZ* mutant cells were independently grown in liquid medium. The cells were incubated overnight, and this cultivation was repeated for 5 to 7 days. Finally, the cultivated cells were plated on L agar plates supplemented with kanamycin. After incubation at 37°C overnight, larger colonies emerged among many smaller colonies. We confirmed that the growth rates of isolated suppressor mutants were faster than those of the *rodZ* mutant cells (Fig. 1A and B). These suppressors were isolated as suppressors of slow-growth phenotype of the *rodZ* mutant (*ssr*) cells. Interestingly, when cells of larger colonies were observed under a microscope, some suppressors showed morphological restoration, and others had recovered a true rod shape (Fig. 1C). To reveal the reason why the rod shape was recovered in the *rodZ* deletion mutant, we further analysed the suppressors.

### Identification of mutation sites of suppressors by whole genome sequencing

To identify a mutation site(s) of the suppressor strains, whole genomic sequencing was carried out using Solexa technology. First, we sequenced the whole genome of the parent strain (BW25113) and compared the sequence of BW25113 with that of W3110 (Accession Number AP009048). Most of the differences between the two strains were derived form insertion sequences (IS) and genotype changes in BW25113. All suppressor strains were found to have a mutation at 2 bp downstream of *rrfG*, which is one of the eight 5S ribosomal RNAs. The mutation did not affect the growth and shape of *ΔrodZ* strains (Shiomi and Niki, 2011). These differences were eliminated from whole genomic sequences of the suppressor strains to identify causal genes of the *ssr* mutants. Suppressor mutations were confirmed genetically by P1 transduction (data not shown). Finally, we found causal mutations in suppressor mutants (Table 1). Of 29 mutants sequenced, 20 suppressors had a mutation in *mreB*, four had a mutation in *mrdA* which encodes PBP2, two had a mutation in *mrdB* which encodes RodA and one had a mutation in the promoter region of *zipA*. We could not identify mutations in the remaining two *ssr* mutants. The majority of mutations occurred in MreB which physically interacts with RodZ. PBP2 and RodA also form a complex with MreB. We further characterized the *ssr* mutations of MreB, PBP2 and RodA in this study.

**Table 1:** Isolated suppressors of slow-growth phenotype of the *rodZ* mutant strain

Mutation site	Replaced by^a^
*mreB*–*Leu17*	Gln (2)
*mreB*–*Asp83*	Tyr (1), Glu (1)
*mreB*–*Arg124*	Cys (2), Ser (3)
*mreB*–*Ala125*	Val (6)
*mreB*–*Arg127*	His (2)
*mreB*–*Glu137*	Ala (1)
*mreB*–*Ala174*	Thr (1)
*mreB*–*Gly329*	Cys (1)
*mrdA*–*Gln51*	Leu (2)
*mrdA*–*Thr52*	Asn (2)
*mrdB*–*Ala234*	Thr (1)
*mrdB*–*Thr249*	Pro (1)

a Number in parenthesis indicates number of clones isolated independently.

### Recovery of the rod shape by *ssr* mutations in MreB

To characterize the phenotypes of the *ssr* mutations of *mreB*, we transferred suppressor mutations to the parent strain (BW25113) or the *rodZ* deletion mutant strain (DS290). In those strains, the chromosomal *mreB* gene was replaced with the *ssr* mutations of *mreB*. These mutations did not affect the cellular concentration of MreB, which was quantified by immunoblotting using anti-MreB antibody (Fig. 1D and E). Of note, the E137A mutation affected the mobility of the MreB protein in SDS-PAGE. All of the *rodZ* deletion mutants carrying *ssr* mutations of *mreB* could grow faster than the *rodZ* deletion mutant carrying wild-type *mreB* in L broth, indicating recovery of active cell growth (Table 2). Thus the *ssr* mutations of *mreB* are sufficient to suppress the slow-growth phenotype of the *rodZ* mutant strain in L broth.

**Table 2:** Mass doubling time (min) of strains carrying suppressor mutations grown in L broth at 37°C

Strains	Mutation(s)	Mass doubling time (min)^a^
BW25113	Wild type	27
DS290	Δ*rodZ*::*kan*	44
DS452	Δ*yhdE*::*cat*	28
DS454	Δ*rodZ*::*kan ΔyhdE*::*cat*	44
DS453	Δ*yhdE*::*cat mreB*–*L17Q*	31
DS455	Δ*rodZ*::*kan* Δ*yhdE*::*cat mreB*–*L17Q*	37
DS552	Δ*yhdE*::*cat mreB*–*D83Y*	26
DS629	Δ*rodZ*::*kan* Δ*yhdE*::*cat mreB*–*D83Y*	35
DS1155	Δ*yhdE*::*cat mreB*–*D83E*	27
DS1156	Δ*rodZ*::*kan* Δ*yhdE*::*cat mreB*–*D83E*	28
DS1159	Δ*yhdE*::*cat mreB*–*R124C*	29
DS1160	Δ*rodZ*::*kan* Δ*yhdE*::*cat mreB*–*R124C*	30
DS1157	Δ*yhdE*::*cat mreB*–*R124S*	27
DS1158	Δ*rodZ*::*kan* Δ*yhdE*::*cat mreB*–*R124S*	28
DS612	Δ*yhdE*::*cat mreB*–*A125V*	26
DS630	Δ*rodZ*::*kan* Δ*yhdE*::*cat mreB*–*A125V*	29
DS1165	Δ*yhdE*::*cat mreB*–*R127H*	28
DS1166	Δ*rodZ*::*kan* Δ*yhdE*::*cat mreB*–*R127H*	34
DS1171	Δ*yhdE*::*cat mreB*–*E137A*	29
DS1172	Δ*rodZ*::*kan* Δ*yhdE*::*cat mreB*–*E137A*	37
DS559	Δ*yhdE*::*cat mreB*–*A174T*	33
DS560	Δ*rodZ*::*kan* Δ*yhdE*::*cat mreB*–*A174T*	37
DS553	Δ*yhdE*::*cat mreB*–*G329C*	28
DS561	Δ*rodZ*::*kan* Δ*yhdE*::*cat mreB*–*G329C*	37
DS674	Δ*rlpA*::*cat*	29
DS675	Δ*rodZ*::*kan ΔrlpA*::*cat*	45
DS685	Δ*rlpA*::*cat mrdA*–*Q51L*	30
DS689	Δ*rodZ*::*kan ΔrlpA*::*cat mrdA*–*Q51L*	38
DS686	Δ*rlpA*::*cat mrdA*–*T52N*	30
DS690	Δ*rodZ*::*kan ΔrlpA*::*cat mrdA*–*T52N*	37
DS687	Δ*rlpA*::*cat mrdB*–*A234T*	30
DS691	Δ*rodZ*::*kan ΔrlpA*::*cat mrdB*–*A234T*	31
DS688	Δ*rlpA*::*cat mrdB*–*T249P*	28
DS692	Δ*rodZ*::*kan ΔrlpA*::*cat mrdB*–*T249P*	32

a Mass doubling time was calculated using absorbance (OD_600_) of cells grown in L broth at 37°C which was automatically recorded by a Bio-photorecorder (TVS 062CA, Advantech) every minute.

**Fig. 2:**
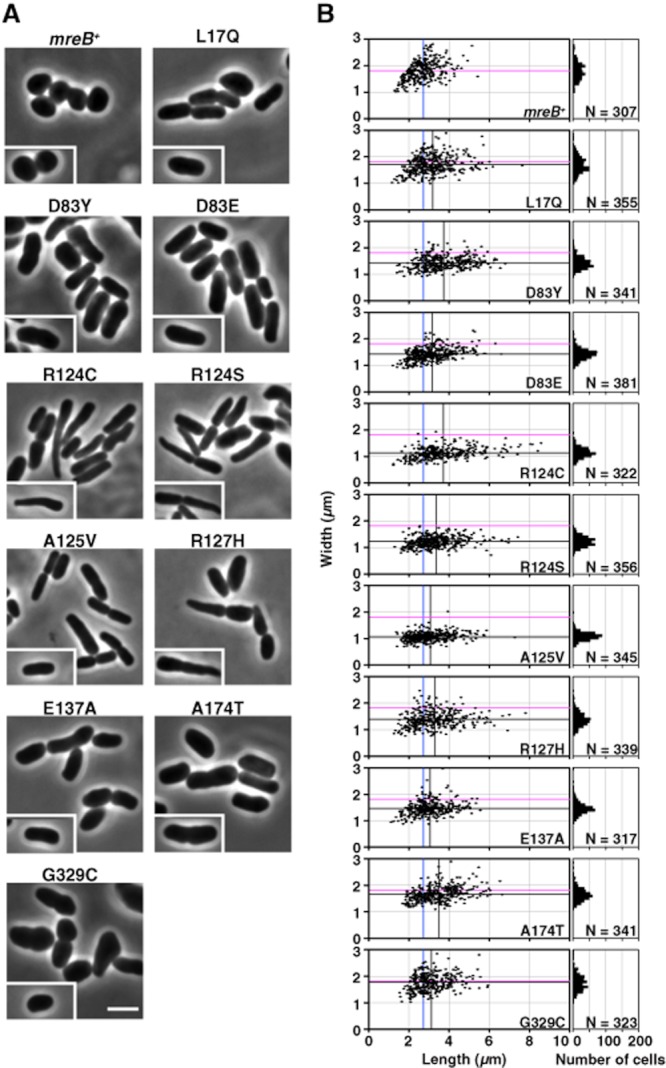
Morphology of Δ*rodZ* cells producing each MreB mutant. A. Phase contrast images of Δ*rodZ* cells producing each MreB mutant. Cells were grown in L broth at 37°C to log phase. Typical cells are shown in inset. Scale bar is 2.5 μm. B. Cell proportions of Δ*rodZ* cells producing each MreB mutant are shown. Distribution of the length and width of each strain is shown (left). Blue and magenta lines indicate average length and width of *mreB*^+^ cells respectively. Black lines indicate average length and width of each strain. Distribution of number of cells to the width is shown (right). Number of cells measured (N) is indicated.

**Fig. 3:**
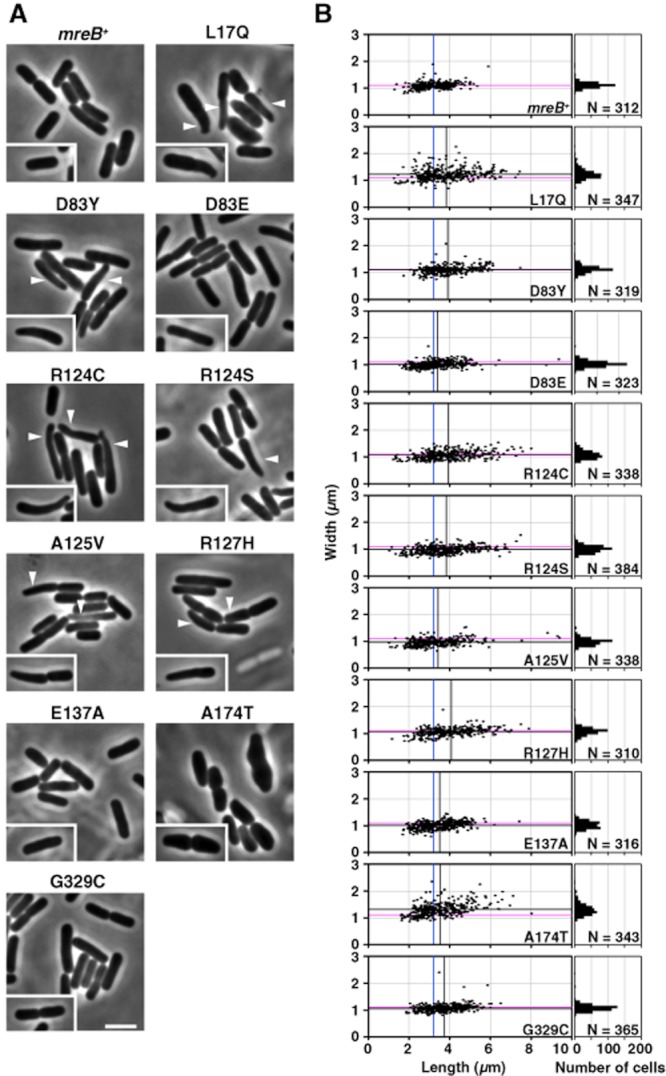
Morphology of *rodZ*^+^ cells producing each MreB mutant. A. Phase contrast images of *rodZ*^+^ cells producing each MreB mutant. Cells are grown in L broth at 37°C to log phase. Typical cells are shown in inset. Scale bar is 2.5 μm. B. Cell proportions of *rodZ*^+^ cells producing each MreB mutant are shown. Distribution of the length and width of each strain is shown (left). Blue and magenta lines indicate average length and width of *mreB*^+^ cells respectively. Black lines indicate average length and width of each strain. Distribution of number of cells to the width is shown (right). Number of cells measured (N) is indicated.

We observed the morphology under the microscope and measured the cell length and cell width of the *ssr* mutants (Figs 2 and 3). The *ssr* mutations of *mreB* improved cell shape in addition to normalizing growth. Cells with the *ssr* mutants became rod shaped in spite of the *rodZ* deletion mutation (Fig. 2). Although the cell width of the *rodZ* deletion mutant is highly variable, the distribution of cell width was relatively narrow in strains carrying R124C, R124S or A125V (Fig. 2). In particular, the cell width of *ΔrodZ* cells producing the MreB–A125V mutant was similar to the wild type (Figs 2 and 3). Thus, the faster growing suppressor mutants of the Δ*rodZ* cells also recovered rod or ovoid cell shapes in every isolate we tested.

To characterize the effect of only the *ssr* mutations on cell morphology, we transferred the *ssr* mutations to the parent strain (BW25113) and observed their morphology under a microscope in order to measure cell length and cell width (Fig. 3). All of the *ssr* mutants with *rodZ* retained their rod shape (Fig. 3). However, the cell width of the *ssr* mutants was affected. Rod cells with MreB–L17Q or MreB–A174T were statistically significantly fatter than wild-type cells (Fig. 3). Roughly 30% of cells producing MreB–L17Q, MreB–D83Y, R124C, R124S, A125V and R127H mutants were tapered as indicated by the arrowheads in Fig. 3A. The tapered cells divided asymmetrically along the long axis as seen in MreB–A125V (inset of Fig. 3A) suggesting that the position of septation was disarranged. Thus, although the *ssr* mutations did not cause a remarkable disruption to cell morphology, the proper regulation of cell width and cell division was affected.

We next examined whether the mutations can suppress the *rodZ* mutation in minimal medium. Although there was an obvious difference in cell growth in minimal medium between wild type and the *rodZ* deletion mutant, the *ssr* mutations could not suppress defects in cell growth except for the MreB–A125V mutation. MreB–A125V mutant cells had partially restored rod shapes regardless of the *rodZ* mutant, but others did not (Fig. S1A). The effect of the *ssr* mutations was dependent on the growing medium.

We selected some of the *ssr* mutations for further study according to the positions of the mutations and their effect on cell width: MreB–L17Q (fatter than MreB–WT in the presence or absence of RodZ, domain IA), D83Y (fatter than MreB–WT in the absence of RodZ, domain IB), A125V (normal rod shape in the absence of RodZ, domain IA), A174T (fatter than MreB–WT in the presence or absence of RodZ, domain IIA) and G329C (fatter than MreB–WT in the absence of RodZ, domain IA) (Figs 2 and 3).

To confirm that *ssr* mutants restored normal geometry (the central cylinder and polar caps), we analysed the distribution of the Tar protein, which localizes to the cell poles (Maddock and Shapiro, 1993), fused with EGFP in wild-type cells. Its aberrant distribution was observed in Δ*rodZ* cells (Fig. S2), where it was distributed around the whole periphery of the cells. On the other hand, Tar–GFP was preferentially localized at the cell poles in DS629 (*ΔrodZ mreB*–*D83Y*), DS630 (*ΔrodZ mreB*–*A125V*) and DS560 (*ΔrodZ mreB*–*A174T*) (Fig. S2). These results suggest that the *ssr* mutants could recover the normal geometry of wild-type cells.

### Mutated residues of MreB that suppress the phenotype of Δ*rodZ*

**Fig. 4:**
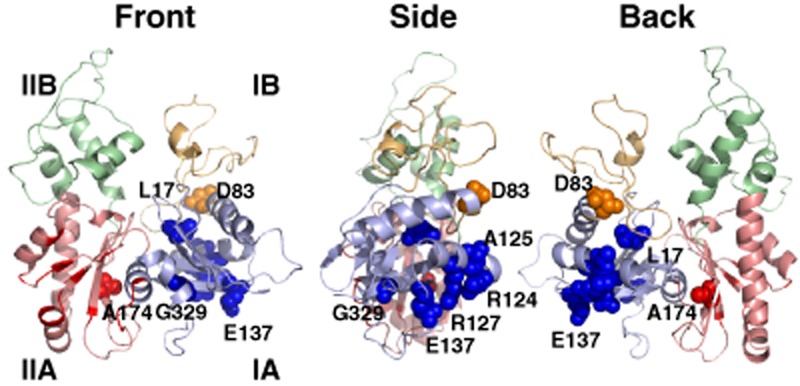
Mapping of the suppressor mutation sites on a 3D structural model of *E. coli* MreB. Each domain is shown in a different colour: IA (cyan), IB (orange), IIA (red) and IIB (green). The spheres show the suppressor mutation sites. Residues coloured dark red in the IIA domain (red) are involved in interaction with RodZ (van den Ent *et al*., 2010). Front, side and back views are shown.

To characterize mutated residues of the MreB structure, we mapped the mutated residues on a three-dimensional structural model of *E. coli* MreB (Fig. 4). The protein structure of MreB of *T. maritima* (PDB: 1JCF) has been determined (van den Ent *et al*., 2001), and so we deduced a three-dimensional structural model of *E. coli* MreB using the modelling program GENO3D (http://geno3d-pbil.ibcp.fr) (Combet *et al*., 2002). Most of the suppressor mutations occurred at residues that were conserved in MreB of *E. coli*, *Caulobacter crescentus*, *B. subtilis* and *T. maritima* (Fig. S3). Furthermore, they were assigned to domain IA which is shown in blue in Fig. 4. One mutation (D83) is located in domain IB, but the position is close to domain IA. Another mutation (A174) was assigned to the middle of the segment of domain IIA and is also located in the vicinity of domain IA in the MreB structural model (Fig. 4).

### Effect of A22 on growth of the *ssr* mutants

**Fig. 5:**
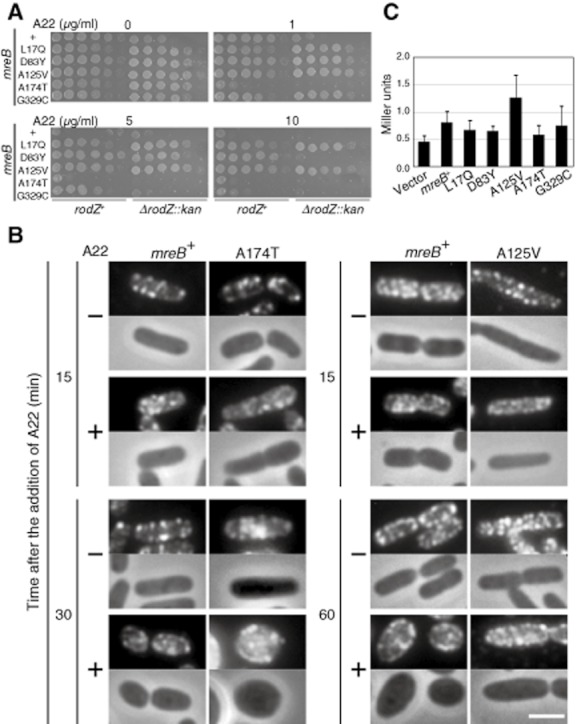
Characterization of MreB mutants. A. Sensitivity of cells producing each MreB mutant to A22. An overnight culture of the indicated strains was diluted serially (from 10^−1^ to 10^−6^) and spotted onto L plates containing A22. The plates were incubated for 24 h at 37°C. B. Immunofluorescent microscopy using anti-MreB antiserum. Cells were harvested at the indicated times after the addition of A22 (left: 1 μg ml^−1^ for cells producing MreB–WT or MreB–A174T; right: 10 μg ml^−1^ for cells producing MreB–WT or MreB–A125V). Fluorescent (top) and phase contrast (bottom) images are shown. Scale bar is 2 μm. C. Properties of self-interaction between MreB mutants determined by the yeast two-hybrid assay. Average of Miller units and error bars (SD) are shown. Twelve independent colonies were examined.

We found that some of the *ssr* mutants were mutated alleles of *mreB*. In general, loss of the *mreB* function causes a defect in cell shape so that rod cells become round cells (Wachi *et al*., 1987). However, the *ssr* mutants of *mreB* maintained a rod-like shape, suggesting that they still retain the biological function of *mreB* in regards to cell morphology. To characterize the function of *mreB* with the *ssr* mutations, we next examined sensitivity to A22, which is an inhibitor of the assembly of *mreB* (Iwai *et al*., 2002) (Fig. 5A and Table 3). BW25113 (WT) could not grow in the presence of more than 5 μg ml^−1^ of A22. On the other hand, growth of DS290 (Δ*rodZ*) was completely inhibited in the presence of more than 1 μg ml^−1^ A22. All *rodZ*^+^ cells carrying *mreB*–*L17Q*, *mreB*–*D83Y*, *mreB*–*A125V* or *mreB*–*G329C* were more resistant to A22 than the wild type. Moreover, these mutations completely (L17Q and A125V) or partially (D83Y and G329C) reduced the sensitivity of the *rodZ* mutant cells to A22. However, the *mreB*–*A174T* mutation did not reduce the sensitivity. On the contrary, this mutation caused the cells to be more sensitive. These results indicate that the *ssr* mutations of MreB affect the assembly of MreB in cells. Thus, we observed the localization of MreB by fluorescence microscopy before and after the addition of A22.

**Table 3:** Summary of properties of *ssr* mutations in MreB

	Cell shape		Sensitivity to A22^a^			
				
Mutation	With RodZ	Without RodZ	With RodZ	Without RodZ	Self-interaction^b^	Interaction with MreC^c^
WT	Rod	Round/oval	++	+++	+	++
L17Q (IA)	Rod^d^	Fat rod/oval	−	−	+	−
D83Y (IB)	Slender^d^	Rod/oval	−	+	+	−
A125V (IA)	Slender^d^	Rod	−	−	++	+++
A174T (IIA)	Short fat	Fat rod/oval	+++	+++	+	+
G329C (IA)	Small rod	Fat rod	+	++	+	+++

a Sensitivity to A22 was judged by viability of each strain on L plate containing A22. +++, highly sensitive; −, resistant.

b Self-interaction was determined by the yeast two-hybrid assay. ++, stronger interaction than WT; +, similar interaction compared with WT.

c Interaction with MreC was determined by the bacterial two-hybrid assay. +++, stronger interaction than MreB–WT; +, weaker interaction than MreB–WT; −, interaction was not detected.

d Cells producing MreB–L17Q, D83Y or A125V were often longer and thinner than wild-type cells.

### Effect of A22 on the assembly of MreB with *ssr* mutations

To further test the sensitivity of A22 in the *ssr* mutants, two *ssr* mutated MreBs were analysed: one was MreB–A125V, which is resistant to A22, and the other was MreB–A174T, which is sensitive. MreB was detected in cells by an anti-MreB antibody. In the absence of A22, MreB–WT, MreB–A125V and MreB–A174T could each be seen as bright and dense spots or spotty patterns along the long axis of the cells (Fig. 5B). Almost no signal of MreB was observed in cytoplasm in all of the strains. Fifteen minutes after the addition of 1 μg ml^−1^ A22, spots or filaments of MreB–WT and MreB–A174T still remained as seen in the absence of A22 and cells producing MreB–WT or MreB–A174T were rod shape. However, 30 min after the addition of 1 μg ml^−1^ A22, cells with MreB–WT or MreB–A174T had changed their rod shapes to ovoid or round. Cells with MreB–A174T were more spherical than those with MreB–WT, which is consistent with the result that the former is more sensitive to A22 than the latter (Fig. 5A). In those cells, MreB–A174T was observed in cytoplasm as well as spots. Because MreB–A125V is more resistant to A22 than the wild type, we used a higher concentration of A22 (10 μg ml^−1^). Spotty or spiral-like patterns of MreB–A125V were still present 15 min after the addition of 10 μg ml^−1^ A22. Wild-type cells changed their cell shape to ovoid or round 60 min after the addition of A22, and MreB were observed as spots and in the cytoplasm. On the other hand, cells with MreB–A125V kept the rod shape although cells became slightly fatter, and the spotty or spiral-like MreB–A125V pattern was clearly retained and some signals were observed in the cytoplasm. This result suggests that the assembly of MreB–A125V could hardly be disturbed by A22. This result is consistent with the result that the growth of *ssr* cells with MreB–A125V is resistant to A22 (Fig. 5A). The *ssr* mutations of MreB provided the ability to keep the rod shape without the RodZ protein because their properties of assembly were modified.

### Self-interaction of MreB with *ssr* mutant proteins

To test whether the differences of the sensitivities to A22 of the *ssr* mutated MreBs were due to self-interaction, we employed a yeast two-hybrid system (Fig. 5C and Table 3). Although the self-interaction of wild-type MreB is weak, it was statistically significantly different from the vector control. Most of the self-interactions of the MreB mutants were comparable to or slightly decreased compared to that of the wild type, while MreB–A125V exhibited a significantly stronger interaction than the wild type. This result indicates that the MreB–A125V mutation affects the self-interaction of MreB, and hence the assembly of MreB filaments. Different properties of self-interaction should affect the morphology of cells.

### Interaction between MreB mutants and MreC

It has been shown an interaction between MreB and MreC by a bacterial two-hybrid assay (Kruse *et al*., 2005). It is possible that the *ssr* mutations of MreB affect the ability of MreB to interact with MreC. To test the binding activity of MreB to MreC, we applied a bacterial two-hybrid as previously reported (Kruse *et al*., 2005). We detected interactions between MreC and MreB–WT, MreB–A125V, MreB–A174T and MreB–G329C when MreC was fused with T25 and when MreB was fused with T18 (Fig. S4A). On the other hand, when MreC was fused with T18 and MreB was fused with T25, interactions between MreC and MreB–A125V or MreB–G329C were detected, although no interaction between MreC and MreB–WT was detected (Fig. S4B), suggesting that these mutations increased the ability of MreB to interact with MreC. We could not detect interactions between MreC and MreB–L17Q or MreB–D83Y, indicating that these mutations compromised the ability of MreB to interact with MreC.

### *ssr* mutations of *mrdA* or *mrdB*

Suppressor mutations were also found in *mrdA* (*mrdA*–*Q51L* and *mrdA*–*T52N*) and *mrdB* (*mrdB*–*A234T* and *mrdB*–*T249P*) genes (Table 1). PBP2, encoded by *mrdA*, plays a role in peptidoglycan synthesis along with RodA, encoded by *mrdB* (Ishino *et al*., 1986). PBP2 is a bitopic membrane protein while RodA is a polytopic membrane protein. Although three-dimensional structures of PBPs from various species have been reported (Sauvage *et al*., 2008), the structure of the domain containing Q51 and T52 has not been solved. Q51 and T52 of *E. coli* PBP2 are located between the transmembrane (TM) domain (residue 13–46) and the dimerization domain (residue 63–240) in the periplasmic domain (Fig. S5A). A234 and T249 of *E. coli* RodA are located between TM6 and TM7 at the periplasmic domain. A234 is highly conserved in most bacteria while T249 is not conserved (Fig. S5B).

### Recovery of rod shape by *ssr* mutations in PBP2 or RodA

**Fig. 6:**
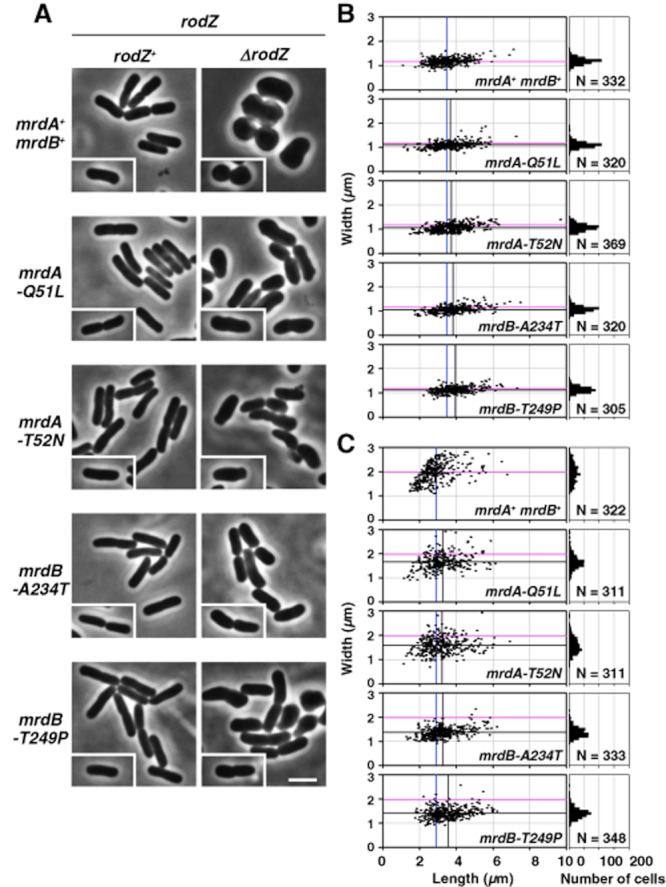
Morphology of *rodZ*^+^ or Δ*rodZ* cells carrying *mrdA* or *mrdB* mutants. A. Phase contrast images of *rodZ*^+^ or Δ*rodZ* cells carrying *mrdA* or *mrdB* mutants. Cells were grown in L broth at 37°C to log phase. Typical cells are shown in inset. Scale bar is 2.5 μm. B and C. Cell proportions of *rodZ*^+^ (B) or Δ*rodZ* (C) cells carrying *mrdA* or *mrdB* mutants. Distribution of the length and width of each strain is shown (left). Blue and magenta lines indicate average length and width of *ssr*^+^ cells respectively. Black lines indicate average length and width of each strain. Distribution of number of cells to the width is shown (right). Number of cells measured (N) is indicated.

We constructed BW25113 (*rodZ^+^*) or DS290 (Δ*rodZ*) strains carrying *mrdA* or *mrdB* mutants. All of the *rodZ* mutant strains carrying either *mrdA* or *mrdB* suppressor mutations grew faster than the *rodZ* mutant strain (Table 2), confirming that these *mrdA* or *mrdB* mutations are suppressors of the slow-growth phenotype of the *rodZ* mutant strain. We next observed the morphology of those strains. *rodZ* mutant cells carrying *mrdA* or *mrdB* mutations had restored rod shapes when grown in L broth, although the cells were slightly fatter than wild-type cells (Fig. 6). These mutations did not alter the morphology of *rodZ^+^* cells. However, these mutations did not restore the rod shape of the *rodZ* mutant in M9 medium (Fig. S1B).

### Effect of antibiotics, mecillinam and A22, on growth of the *mrdA* or *mrdB* mutants

**Fig. 7:**
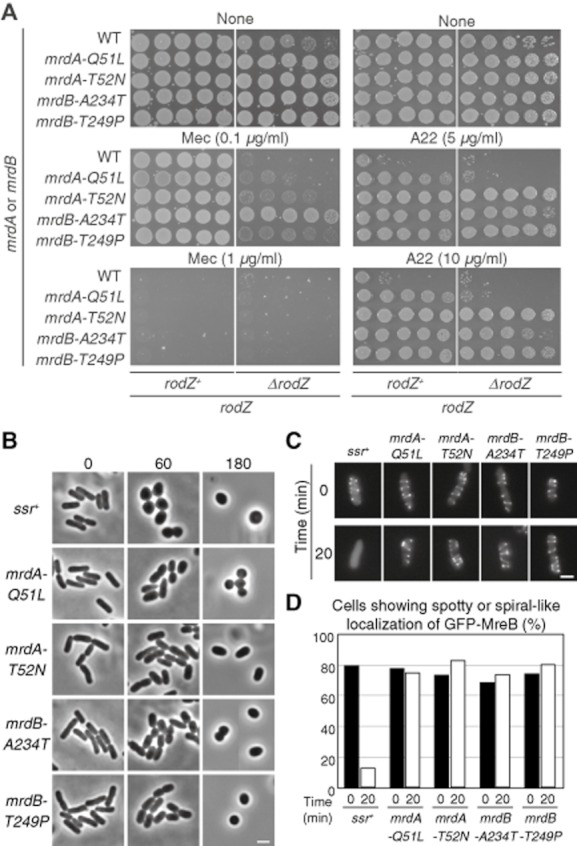
Characterization of PBP2 and RodA mutants. A. Sensitivity of cells carrying *mrdA* or *mrdB* mutations to mecillinam and A22. Overnight cultures of the indicated strains were diluted serially (from 10^−1^ to 10^−5^) and spotted onto L plates containing mecillinam or A22. The plates were incubated for 24 h at 37°C. B. Phase contrast images of cells at indicated time points after the addition of 10 μg ml^−1^ A22. Scale bar is 2 μm. C. Typical fluorescent images of cells showing spotty or spiral-like localization of GFP–MreB in DS797 (WT), DS798 (*mrdA*–*Q51L*), DS1324 (*mrdA*–*T52N*), DS799 (*mrdB*–*A234T*) or DS1325 (*mrdB*–*T249P*). *gfp*–*mreB* was expressed from a plasmid (pDS176) and induced by 10 μM sodium salicylate. Cells were grown to log phase and 1 μg ml^−1^ A22 was added to the culture (time 0). Then cells were harvested at 20 min after the addition of A22 and observed. Scale bar is 2 μm. D. Proportion (%) of cells showing spotty or spiral-like localization of GFP–MreB. More than 300 cells were counted.

The suppressor mutation sites in PBP2 and RodA are located in the periplasmic domain, which is involved in synthesis of the peptidoglycan layer at the central cylindrical part of the rod cell. It is thought that these mutations change the structure of the active site or activity of PBP2, and thus the mutations would affect the ability of PBP2 to bind to beta-lactam antibiotics. Thus, we examined the sensitivity of cells carrying the *ssr* mutations of *mrdA* or *mrdB* to mecillinam, a specific inhibitor of PBP2. As seen in Fig. 7A, DS290 (Δ*rodZ*) was more sensitive to mecillinam than BW25113. The sensitivities of *rodZ*^+^ cells carrying mutations in *mrdA* or *mrdB* to mecillinam were comparable to that of the parent strain (*rodZ*^+^). The *rodZ*-deleted cells carrying the *ssr* mutations of *mrdA* or *mrdB* were only slightly resistant to mecillinam compared with the *rodZ* mutant (Fig. 7A), suggesting that the mutations did not significantly affect the structure of the active site of PBP2, but possibly altered other cellular processes to bypass the function of RodZ.

We then tested the sensitivity of these strains to A22, which disrupts MreB filaments (Fig. 7A). Surprisingly, all of the strains were more resistant to A22 than the wild-type strain, even though all of the strains carried wild-type MreB. We also observed the cell shapes of the *ssr* strains after the addition of A22 (Fig. 7B). All of the *ssr* mutants still showed the rod shape, while wild-type cells were round or oval 1 h after the addition of A22. We also examined the localization of GFP–MreB in the *ssr* mutants (Fig. 7C and D). Although all *ssr* strains were rod-shaped, GFP–MreB was diffused in the cytoplasm in DS797 (WT) 20 min after the addition of A22, while spots or spiral patterns of GFP–MreB were still present in DS798 (*mrdA*–*Q51L*), DS1324 (*mrdA*–*T52N*), DS799 (*mrdB*–*A234T*) or DS1325 (*mrdB*–*T249P*), indicating that the assembly of MreB in the *ssr* mutants is more stable than in wild-type cells. Because MreB and PBP2/RodA form a complex, these results suggest that the *ssr* mutations of *mrdA* or *mrdB* affect the dynamics of the MreB polymers or the integrity of the complex.

## Discussion

We isolated fast growing cells, assuming them to be spontaneous suppressor mutants, among populations of the *rodZ* deletion strain. All *ssr* suppressor mutants had lost the round shape and had recovered their rod shape or a rod-like shape. We previously pointed out that a change of cell shape not only affects the structure of the cell membrane, it also affects the metabolic activities inside the cell because of the reduction in the ratio of cell surface area to cell volume (Shiomi *et al*., 2009). The reduction of the ratio of cell surface area to volume in rod cells compromises the metabolic activity of the cell membrane (Ito *et al*., 2005; Hara *et al*., 2009). Naturally, fast growth of the *rodZ* mutant should be coincident with recovery of the cell shape.

### Function of domain IA of MreB in rod-shaped cells

As RodZ functions with MreB to maintain the rod shape of *E. coli*, it makes sense that most suppressor mutations of the *rodZ* mutant map to the *mreB*, *mrdA* or *mrdB* genes, which are additional components required for synthesis of peptidoglycan. These mutants can bypass the loss of function of RodZ to maintain rod-shaped cells, suggesting that RodZ regulates the functions of these gene products rather than being an essential component. Of note, the *ssr* mutations in MreC and MreD which form complex with MreB have not been isolated so far.

A co-crystal structure of *T. maritima* MreB and the cytoplasmic domain of *T. maritima* RodZ has been solved (van den Ent *et al*., 2010). Based on the structure of *T. maritima* MreB, we can hypothesize how *ssr* mutations of MreB compensate for the loss of function of RodZ to maintain rod-shaped cells. First, all of the *mreB* suppressor mutations were mapped to or proximate to domain IA of MreB. Although all four domains of MreB are likely to contribute to self-interaction of MreB in some fashion, the roles of domain IA of MreB or domain 1 of actin have not yet been well characterized. Our results suggest possible roles of this domain. First, domain IA contributes to the self-interaction of MreB and hence would affect the assembly and dynamics of MreB filaments. Another possible role of domain IA of MreB would be as an interface for binding to RodZ. However, the co-crystal structure of MreB and the cytoplasmic domain of RodZ from *T. maritima* reveals that the residues of MreB involved in interacting with RodZ are in domain IIA, which is on the complete opposite face in the MreB structure (Fig. 4) (van den Ent *et al*., 2010). Even though the RodZ binding site in domain IIA of MreB is distant from domain IA, it is possible that domain IIA of MreB is close to domain IA in another MreB filament if MreB forms two-stranded helical filaments *in vivo* as it does *in vitro* (van den Ent *et al*., 2001; Salje *et al*., 2011). A third possible role of the domain is as an interface for binding to other proteins including MreC. In fact, some *ssr* mutations increased or decreased the ability of MreB to interact with MreC (Fig. S4).

Most of the MreB suppressors are located on the outside of the MreB molecule and are more resistant to A22 than the wild type. There are three possible types of filamentous structures of MreB strands: pairs of antiparallel protofilaments, sheets of parallel protofilaments and pairs of parallel protofilaments (Salje *et al*., 2011) (Fig. S6). In any arrangement of MreB protofilaments, these mutation sites are located at the lateral contact between each protofilament so that they might affect the interaction between the protofilaments for their stabilization. In addition, these suppressor mutations are close to the cytoplasmic membrane so that they should be able to interact with the relatively short cytoplasmic region of MreC (nine residues for *E. coli* MreC).

### Cell width control

It has been reported that cells producing MreB–D165A/E/V change the cell width (Kruse *et al*., 2003). MreB–D165A/E/V mutants are non-functional and cells producing MreB–D165A/E/V are not viable. Ectopic production of MreB–D165 mutants in wild-type cells perturbs the formation of MreB filaments. In addition, *B. subtilis* MreB–D158A (a corresponding mutant to *E. coli* MreB–D165A) forms filaments, but the filaments are less dynamic than MreB–WT (Defeu Soufo and Graumann, 2006). Cells producing MreB*_Bs_*–D158A are slightly wider (Defeu Soufo and Graumann, 2006). The morphology of MreB–D165A/E/V is very much similar to that of cells producing MreB–A174T (Fig. 2), although cells producing MreB–A174T are viable while cells producing MreB–D165A/E/V are not. On the other hand, some of the *mreB* mutants, such as *mreB*–*D83Y* and *mreB*–*A125V*, were thinner than the parent strain. Because cells lacking *rodZ* are also wider than those of the parent strain (Shiomi *et al*., 2008; Alyahya *et al*., 2009; Bendezu *et al*., 2009), it is thought that a correct interaction between MreB and RodZ is important for width control. If so, cells producing MreB–A125V are able to synthesize peptidoglycan without RodZ, but are not able to accurately regulate cell width because of a lack of RodZ.

The thinner mutants, such as MreB–A125V, can be used for other biological studies. For example, cryo-electron tomography (cryo-ET) has been used to observe the three-dimensional structures of cells and subcellular complexes in their native and frozen-hydrated states at nanometer resolution. However, *E. coli* cells are relatively thick so that the bacterium is not suitable for cryo-EM. Cells producing MreB–A125V are significantly thinner than the parent strain (Fig. 3). This mutant has been used to observe how bacteriophage P1 infects *E. coli* by cryo-ET (Liu *et al*., 2011).

### Mechanism of restoration of the rod shape without RodZ

We previously hypothesized that RodZ determines the cell length by regulating peptidoglycan synthesis (Shiomi *et al*., 2008; 2009). In this study, most of the suppressor mutations were mapped to the *mreB*, *mrdA* and *mrdB* genes. So how do these mutants maintain their rod shape without RodZ? It is thought that MreB regulates the subcellular localization of peptidoglycan synthesis enzymes such as PBP2 and RodA (Figge *et al*., 2004; Kruse *et al*., 2005; Leaver and Errington, 2005; Divakaruni *et al*., 2007; Kawai *et al*., 2009). Therefore, the simplest idea is that MreB mutants that form firm filaments without RodZ would be able to properly place peptidoglycan synthesis enzymes. In support of this, cells producing MreB–L17Q, D83Y, A125V or G329C mutants are more resistant to A22, suggesting that these MreB mutants can form stable polymers even without RodZ, although we could not detect a significant increase in the self-interaction of MreB, except for MreB–A125V. Alternatively, these MreB mutants could form stable polymers due to a complex with MreC and MreD regardless of loss of RodZ. It is known that MreB forms a complex with MreC and MreD, and MreC interacts with MreB in bacterial two-hybrid assay (Kruse *et al*., 2005; Leaver and Errington, 2005; van den Ent *et al*., 2006). In fact, MreB–A125V and MreB–G329C bind to MreC more strongly than does MreB–WT.

On the other hand, MreB–L17Q and MreB–D83Y did not increase an ability of self-interaction and rather decreased an ability to interact with MreC. In this case, these mutations provably increase an ability to interact with other proteins such as PBP2 or RodA.

Cells producing MreB–A174T were more sensitive to A22 than wild-type cells. A174 is involved in the phosphate 2 domain (Bork *et al*., 1992; Kruse *et al*., 2003). In general, a mutation in a conserved residue in phosphate 2 results in a strong reduction of ATPase activity (Bork *et al*., 1992; Kabsch and Holmes, 1995; Posern *et al*., 2002). Thus, the MreB–A174T mutation may change the ATPase activity or simply decrease the ability of ATP binding. In any case, MreB–A174T would decrease the ability of self-interaction as detected by yeast two-hybrid assay although it was very slight decrease. It has been recently shown that MreB–D158A, believed to inhibit ATP hydrolysis, displays circumferential movements at speeds similar to those observed with MreB–WT (Garner *et al*., 2011). Even though the self-interaction of MreB–A174T is weaker than that of MreB–WT, it should still retain the ability to move. This movement might take place independently of RodZ, and thus cells would be able to synthesize cylindrical peptidoglycan without RodZ.

PBP2 and RodA, encoded by *mrdA* and *mrdB* respectively, may indirectly affect the assembly of MreB. This is suggested by our results showing that cells carrying *mrdA* or *mrdB* mutants are more resistant to A22 than wild-type cells (Fig. 7).

Interestingly, the suppressor mutations except for *mreB*–*A125V* could not restore the cell shape of the *rodZ* mutant in minimal medium, indicating that most MreB, PBP2 and RodA mutants are not sufficient to bypass the absence of RodZ in minimal medium. Because the *ssr* mutants were isolated in cells grown in L broth, it is not surprising that the mutations do not necessarily function in minimal medium as they do in rich medium. Because MreB–A125V is the only mutation whose self-interaction is stronger than that of MreB–WT (Fig. 5C), it is possible that strong MreB filaments can restore cell shape of the *rodZ* mutant in minimal medium.

It has been reported that selected isolates of motile pseudo-revertants of the *rodZ* deletion mutant recover their swarming ability (Niba *et al*., 2010). The morphology of these revertants is reminiscent of our suppressors. Indeed, some of our *ssr* mutants could restore the swarming ability of the *rodZ* mutant (Fig. S7). The authors of that study did not characterize the revertant mutation sites and concluded that the *rodZ* gene is not essential for cell shape maintenance. However, a suppressor mutation is certainly needed to recover the rod shape of fast growing cells.

### Suppression of a cold-sensitive phenotype of the *rodZ* mutant

It has been shown that the *rodZ* mutant exhibits a cold-sensitive phenotype (Bendezu *et al*., 2009), although the mechanism is still unknown. We have shown that a mutation in the *ispA* gene suppresses the cold-sensitive phenotype but does not restore the rod shape (Shiomi and Niki, 2011). IspA is involved in the formation of farnesyl pyrophosphate in the isoprenoid biosynthesis pathway (Fujisaki *et al*., 1990). This pathway then contributes to the formation of several essential lipids involved in peptidoglycan synthesis. Because the amount of peptidoglycan is reduced in the *rodZ* mutant (Niba *et al*., 2010), the integrity of the cell wall, including peptidoglycan, may be reduced. The reduction of integrity of the cell wall may result in cold sensitivity in the mutant. We also found that our *ssr* mutations suppressed the cold-sensitive phenotype of the mutant (Fig. S8). Therefore, it is a plausible hypothesis that our *ssr* mutants restore the amount of peptidoglycan in the *rodZ* mutant as well as the cell shape and growth rate.

### Conclusions

It is very likely that RodZ regulates the assembly of MreB and hence regulates peptidoglycan synthesis to determine cell shape. A change of cell shape affects the integrity of the cell wall, cell membrane and cellular metabolism. Thus the cell shape and cell size are significant factors for adaptive proliferation of bacteria in their particular environments. RodZ, which is widely conserved in bacteria, might contribute to fine-tuning these factors in each bacterial species.

## Experimental procedures

### Bacterial strains and growth media

All strains are derivatives of *E. coli* K-12 and are listed in Table S1. BW25113 (Datsenko and Wanner, 2000) is a wild-type strain. DS290 (BW25113 Δ*rodZ*::*kan*) was constructed by P1 transduction, transferring Δ*rodZ*::*kan* prepared from JW2500 (Δ*rodZ*::*kan*) (Shiomi *et al*., 2008) to BW25113. Cells were grown in L broth (1% bacto tryptone, 0.5% yeast extract, 0.5% NaCl) at the indicated temperature. Kanamycin (15 μg ml^−1^), ampicillin (100 μg ml^−1^) and chloramphenicol (20 μg ml^−1^) were added to the culture medium when necessary.

### Plasmids

All plasmids used in this study are listed in Table S2. *rodZ* and *mreB* genes were amplified using BW25113 cells as a template and high fidelity DNA polymerase KOD-plus (TOYOBO). The PCR products were cloned as SacI–HindIII and NdeI–HindIII fragments into pET28a to yield pDS725 (pET28a–*his_6_*–*mreB*) and pDS522 (pET28a–*his_6_*–*rodZ_1–111_*) respectively. The PCR products of *mreB* and mutated genes were cloned as NdeI–BamHI fragments into pGADT7 or pBKT7 to yield plasmids for the yeast two-hybrid system.

### Isolation of suppressors

Independent single colonies of DS6 were grown in L broth containing kanamycin (15 μg ml^−1^) at 37°C. Every day or 2 days, cells were transferred to fresh L broth containing kanamycin and kept growing at 37°C. Five to 7 days after shaking cells, cells were plated on L plates containing kanamycin (15 μg ml^−1^) and incubated at 37°C. Bigger colonies were isolated as suppressors of the slow-growth phenotype of the *rodZ* mutant.

### Genome sequencing

Chromosomal DNAs of wild-type and suppressor strains were extracted and sequenced by the Solexa system.

### Construction of strains carrying suppressor mutations

To transfer suppressor mutations, PCR fragments containing a cat (chloramphenicol acetyltransferase) cassette flanked by a FLP recognition target site were inserted between the first and second codon of chromosomal genes, which were downstream of the gene of interest, in each suppressor strain carrying the λ Red expression plasmid pKD46 (Datsenko and Wanner, 2000). pKD3 was used as a template for PCR. To transfer *mreB*, *mrdA* and *mrdB* mutations, chloramphenicol resistance (Cm^R^) colonies were isolated after transformation of suppressors, which have mutations in *mreB*, *mrdA* or *mrdB*, with PCR fragments to insert a cat resistance cassette in the *yhdE* gene which is downstream of *mreD* (for *mreB* mutations), and in the *rlpA* gene, which is downstream of *mrdB* (for *mrdA* and *mrdB* mutations). P1 phage was grown on a donor that carried *mreB* mutations and the *yhdE* gene inserted with a cat resistance cassette, or *mrdA*/*mrdB* mutations and the *rlpA* gene inserted with a cat resistance cassette, and were used to transduce BW25113 (wild type) or DS290 (Δ*rodZ*::*kan*). Fresh transductants were restreaked on L plates containing Cm, and we selected Cm^R^ clones. All of the mutation sites were sequenced and confirmed. To remove the cat cassette, these strains were transformed with pCP20 at 30°C, and then the transformants were incubated at 42°C. Cells sensitive to Cm were selected.

### Anti-MreB antiserum production

To raise antiserum against MreB, a 6× His (His_6_) tag was added to the N-terminus of MreB. KRX carrying pDS725 (a plasmid encoding *his_6_*–*mreB*) was grown in L broth supplemented with Kan and 0.25% glucose overnight. Cells were diluted 100 times into fresh L broth supplemented with Kan and incubated for 3 h at 37°C. Then cells were transferred to 25°C and incubated for 30 min. 0.1% rhamnose was added to the culture and the cells were incubated at 25°C overnight. Cells were harvested by centrifugation and resuspended in Buffer A [50 mM NaPO_4_ (pH 7), 300 mM NaCl, 8 M urea] supplemented with one tablet of EDTA-free protease inhibitor cocktail (Roche). The mixture was incubated for 1 h at 37°C, and centrifuged at 12 000 r.p.m. for 20 min at 4°C. The supernatant containing His_6_–MreB was mixed with Talon metal affinity resin (Clontech) equilibrated by Buffer A. The mixture was incubated at 4°C overnight. The mixture was centrifuged and the supernatant removed. The resin was resuspended in Buffer A and centrifuged. This step was repeated three times. His_6_–MreB was eluted by Buffer A containing 150 mM imidazole. The protein was used to immunize rabbits, and anti-MreB antiserum was produced by Biogate (Gifu, Japan).

### Immunoblotting

Immunoblotting was performed with a nitrocellulose membrane, Hybond-ECL (GE Healthcare) using anti-MreB antiserum.

### Microscopy

Cells were grown at 37°C in L broth overnight. The culture was diluted in L broth and exponentially grown at 37°C. To observe cells, equal amounts of cells were mixed with 2% agarose. For immunofluorescent microscopy using anti-MreB antiserum, cells were dropped on a slide glass coated by poly-lysine and incubated for 15 min. Cells that were not fixed onto a slide glass were discarded. The glass slide was placed in fixation solution (methanol : acetic acid : H_2_O = 3:1:4) and incubated overnight. The slide glass was placed in PBS for 10 min. Thirty microlitres of lysozyme (8 mg ml^−1^) was dropped onto the slide glass, which was then incubated for 10 min and placed in PBS. Thirty microlitres of 3% BSA was then dropped onto it, and then it was incubated for 10 min after which 30 μl of anti-MreB antiserum diluted 100 times with 3% BSA was dropped onto it. The slide glass was then incubated for 1 h at RT, then it was placed in PBS for 10 min and 30 μl of goat anti-rabbit antibody conjugated with Alexa Fluor 488 (Molecular Probe) diluted 100 times with 3% BSA was dropped onto it. The slide was then incubated for 3 h at RT in the dark, after which it was placed in PBS for 10 min and observed. The cells were observed under an epifluorescence microscope (Zeiss, AXIO) and images were processed by Adobe Photoshop CS4. To measure the lengths of the long and short axes of cells, digital images were processed by the software Metamorph and changed into binary images to detect cell outlines. Each cell in the binary images was automatically measured for the length of the longest line (the long axis of the cell) and maximum breadth perpendicular to the longest line (the short axis of the cell). All experiments were repeated at least twice. *P*-value was determined by unpaired *t*-test and *P*-value < 0.05 was considered significantly different.

### Yeast two-hybrid assay

Plasmids pGADT7 and pGBKT7 carrying each *mreB* mutant were used to transform yeast strain AH109. The protocol to measure β-galactosidase activity (Miller units) was provided by Clontech.

### Bacterial two-hybrid assay

Plasmids encoding *T25*–*mreC* and *T18*–*mreB*, or *T25*–*mreB* and *T18*–*mreC*, were used to transform BTH101 (Karimova *et al*., 1998). The transformants were streaked on P plates (1% Bacto-peptone, 0.5% NaCl, 1.5% agar) containing X-gal (40 μg ml^−1^).
