# Supplementary material for: Mutations in cell elongation genes mreB, mrdA and mrdB suppress the shape defect of RodZ-deficient cells
Source: Mol Microbiol. 2013 Jan 21;87(5):1029–44. doi: 10.1111/mmi.12148 (PMC3599482; doi:10.1111/mmi.12148)
Supplement: Supplementary file 1 [file mmi0087-1029-SD1.pdf]

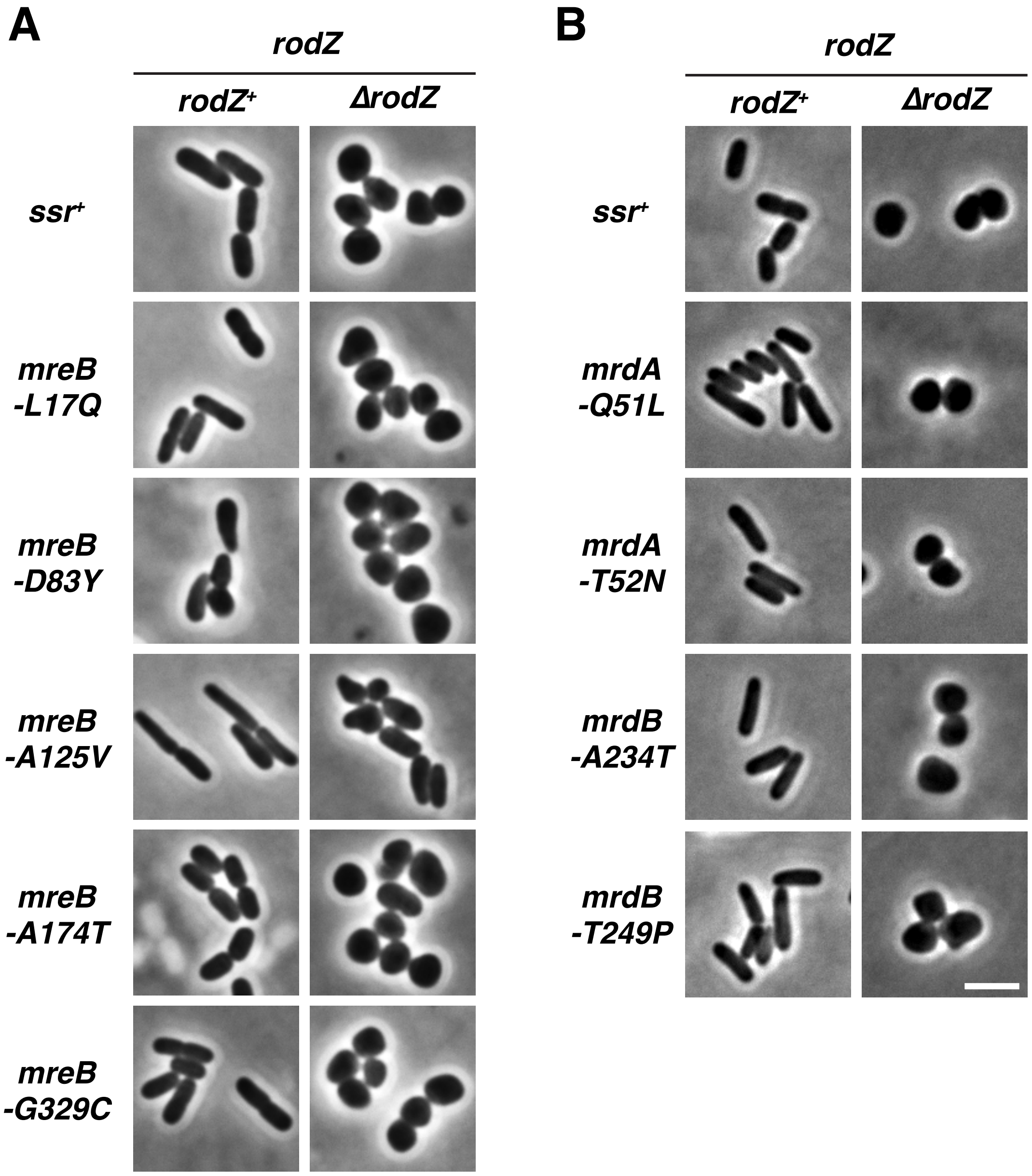

Supplementary Figure S1 Shiomi et al,

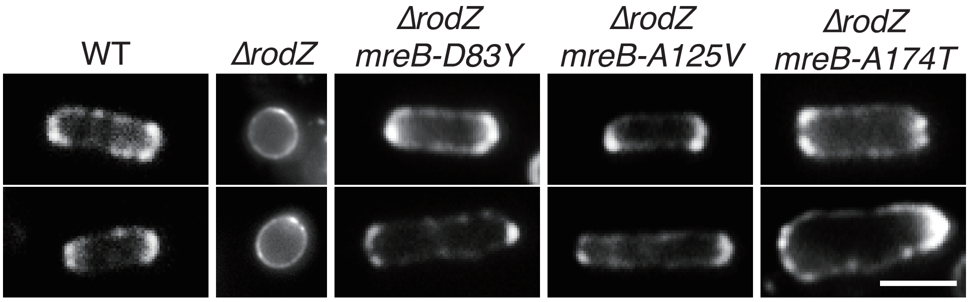

Supplementary Figure S2 Shiomi et al,

```
CLUSTAL 2.0.12 multiple sequence alignment

                                     17
MreB_Ecoli              MLKKFRGMFSNDLSIDLGT-ANTLIYVKGQGIVLNEPSVVAIRQDRAGSPKSVAAVGHDA 59
MreB_Ccrescentus        MFSSLFGVISNDIAIDLGT-ANTLIYQKGKGIVLNEPSVVALRN--VGGRKVVHAVGIEA 57
MreB_Bsubtilis          ----MFGIGARDLGIDLGT-ANTLVFVKGKGIVVREPSVVALQT----DTKSIVAVGNDA 51
MreB_Tmaritima          -------MLRKDIGIDLGT-ANTLVFLRGKGIVVNEPSVIAIDS----TTGEILKVGLEA 48
Actin_Scerevisiae       -----MDSEVAALVIDNGSGMCKAGFAGDDAPRAVFPSIVGRPR----HQGIMVGMGQKD 51

                                                  83
MreB_Ecoli              KQMLGRTP---GNIAAIRPMKDGVIADFFVTEKMLQHFIKQVHSN-SFMRPSPRVLVCVP 115
MreB_Ccrescentus        KQMLGRTP---GHMEAIRPMRDGVIADFEVAEEMIKYFIRKVHNRKGFVN--PKVIVCVP 112
MreB_Bsubtilis          KNMIGRTP---GNVVALRPMKDGVIADYETTATMMKYYINQAIKNKGMFARKPYVMVCVP 108
MreB_Tmaritima          KNMIGKTP---ATIKAIRPMRDGVIADYTVALVMLRYFINKAKGGMNLFK--PRVVIGVP 103
Actin_Scerevisiae       SYVGDEAQSKRGILTLRYPIEHGIVTNWDDMEKIWHHTFYNELRVAPEEH--PVLLTEAP 109

                           124/125 127      137                                174
MreB_Ecoli              VGATQVEERRAIRESAQGAGAREVFLIEEPMAAAIGAGLPVSEATGSMVVDIGGGTTEVAV 175
MreB_Ccrescentus        SGATAVERRAINDSCLNAGARRVGLIDEPMAAAIGAGLPIHEPTGSMVVDIGGGTTEVAV 172
MreB_Bsubtilis          SGITAVERRAVIDATRQAGARDAYPIEEPFAAAIGANLPVWEPTGSMVVDIGGGTTEVAI 168
MreB_Tmaritima          IGITDVERRAILDAGLEAGASKVFLIEEPMAAAIGSNLNVEEPSGNMVVDIGGGTTEVAV 163
Actin_Scerevisiae       MNPKSNREKMTQIMFETFNVPAFYVSIQAVLSLYSSGR-----TTGIVLDSGDGVTHVVP 164

MreB_Ecoli              ISLNGVVYSSSVRIG--GDRFDEAIINYVRR---NYGSLIGEATAERIKHEIGSA-YPGD 229
MreB_Ccrescentus        LSLSGIVYSRSVRVG--GDKMDEAIISYMRR---HHNLLIGETTAERIKKEIGTARAPAD 227
MreB_Bsubtilis          ISLGGIVTSQSIRVA--GDEMDDAIINYIRK---TYNLMIGDRTAEAIKMEIGSAEAPEE 223
MreB_Tmaritima          ISLGSIVTWESIRIA--GDEMDEAIVQYVRE---TYRVAIGERTAERVKIEIGNVFPSKE 218
Actin_Scerevisiae       IYAGFSLPHAILRIDLAGRDLTDYLMKILSERGYSFSTTAEREIVRDIKEKLCYVALDFE 224

MreB_Ecoli              -EVREIEVRG-----RNLAEGVPRGFTLNSNEILEALQEPLTGIVSAVMVALEQCPPELA 283
MreB_Ccrescentus        GEGLSIDVKG-----RDLMQGVPREVRISEKQAADALAEPVGQIVEAVKVALEATPPELA 282
MreB_Bsubtilis          --SDNMEIRG-----RDLLTGLPKTIEITGKEISNALRDTVSTIVEAVKSTLEKTPPELA 276
MreB_Tmaritima          NDELETTVSG-----IDLSTGLPRKLTLKGGEVREALRSVVVAIVESVRTTLEKTPPELV 273
Actin_Scerevisiae       QEMQTAAQSSSIEKSYELPDGQVITIGNERFRAPEALFHPSVLGLESAGIDQTTYNSIMK 284

                                                                           329
MreB_Ecoli              SDISER-----GMVLTGGGALLRNLDRLLMEET--------GIPVVVAEDPLTCVARGGG 330
MreB_Ccrescentus        SDIADK-----GIMLTGGGALLRGLDAEIRDHT--------GLPVTVADDPLSCVALGCG 329
MreB_Bsubtilis          ADIMDR-----GIVLTGGGALLRNLDKVISEET--------KMPVLIAEDPLDCVAIGTG 323
MreB_Tmaritima          SDIIER-----GIFLTGGGSLLRGLDTLLQKET--------GISVIRSEEPLTAVAKGAG 320
Actin_Scerevisiae       CDVDVRKELYGNIVMSGGTTMFPGIAERMQKEITALAPSSMKVKIIAPPERKYSVWIG-G 343

MreB_Ecoli              KALEMIDMHGGDLFSEE--------------- 347
MreB_Ccrescentus        KVLEHPKWMKGVLESTLA-------------- 347
MreB_Bsubtilis          KALEHIHLFKGKTR------------------ 337
MreB_Tmaritima          MVLDKVNILKKLQGAG---------------- 336
Actin_Scerevisiae       SILASLTTFQQMWISKQEYDESGPSIVHHKCF 375
```

Supplementary Figure S3 Shiomi et al,

A **B**

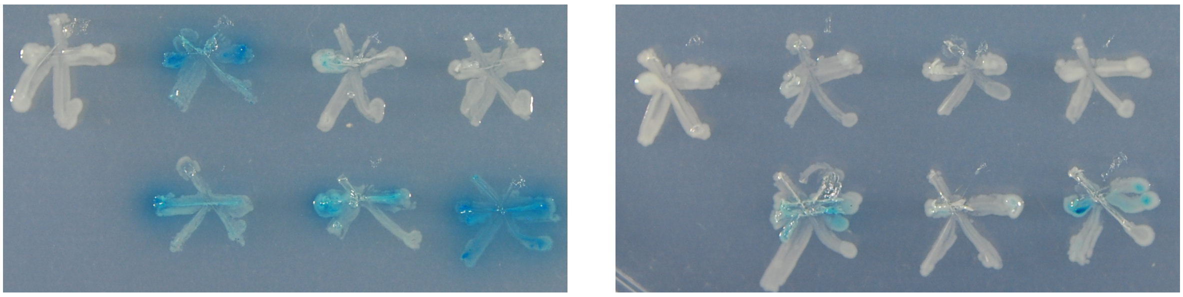

	A				B			
T25-	none	*mreC*	*mreC*	*mreC*	none	*mreB*	*mreB-L17Q*	*mreB-D83Y*
T18-	none	*mreB*	*mreB-L17Q*	*mreB-D83Y*	none	*mreC*	*mreC*	*mreC*
		mreC	*mreC*	*mreC*		*mreB-A125V*	*mreB-A174T*	*mreB-G329C*
		mreB-A125V	*mreB-A174T*	*mreB-G329C*		*mreC*	*mreC*	*mreC*

Supplementary Figure S4 Shiomi et al,

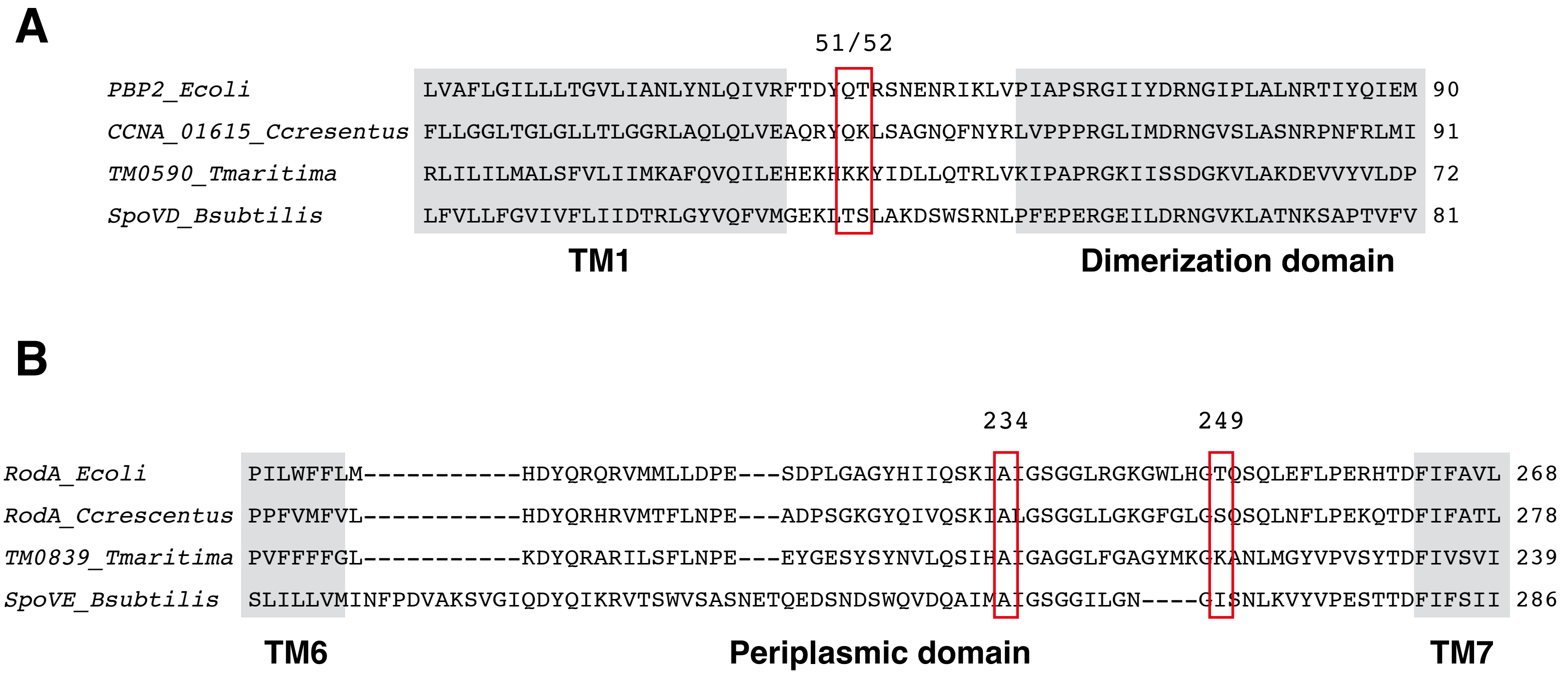

Supplementary Figure S5 Shiomi et al,

A antiparallel

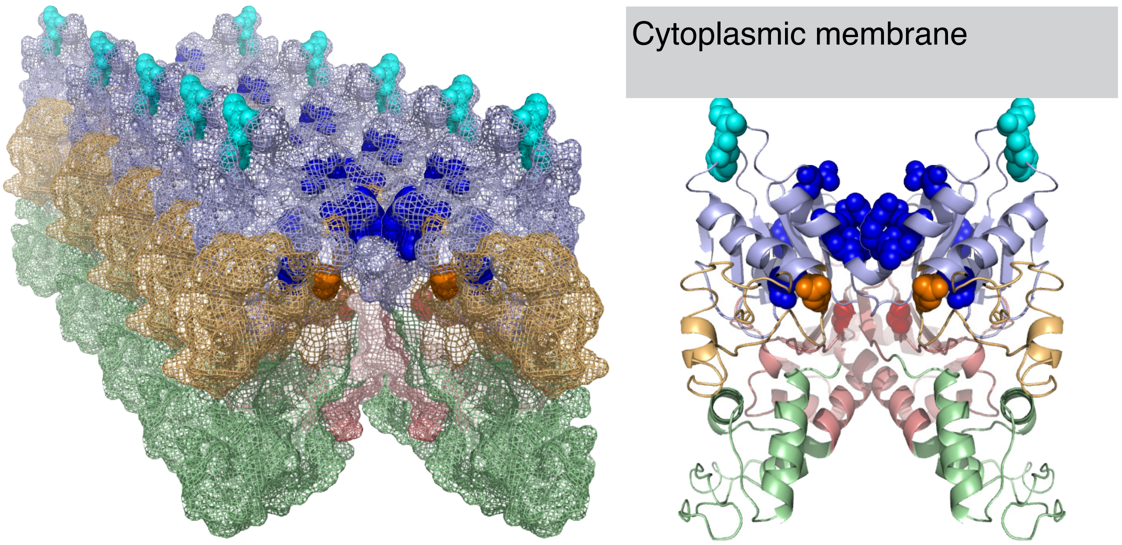

B parallel

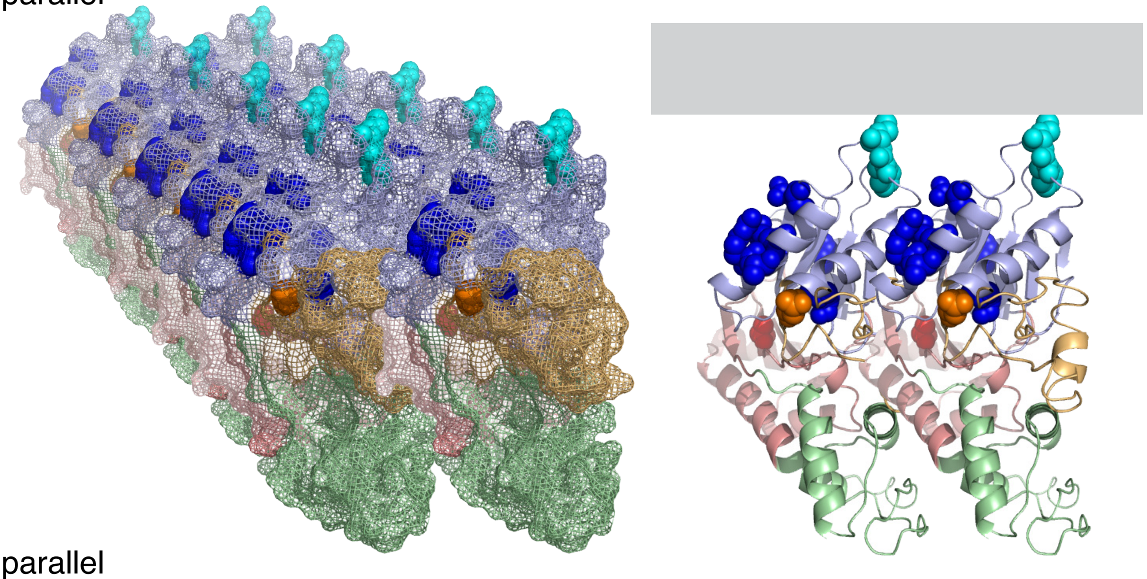

C parallel

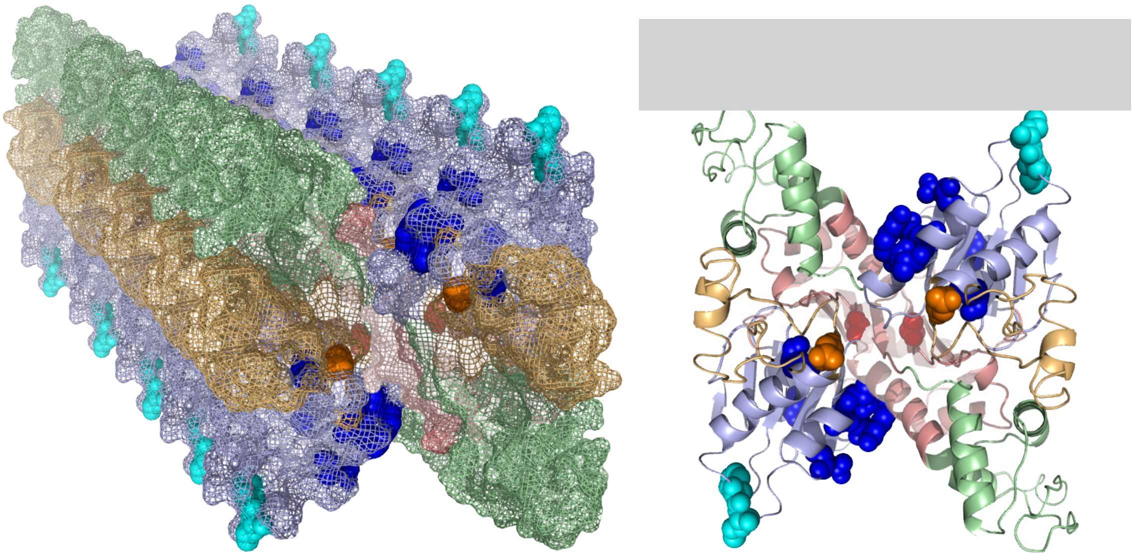

Supplementary Figure S6 Shiomi et al,

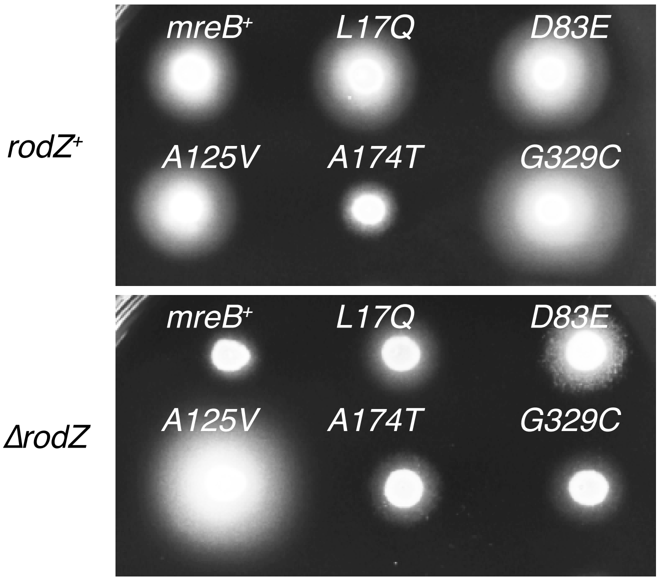

Supplementary Figure S7 Shiomi et al,

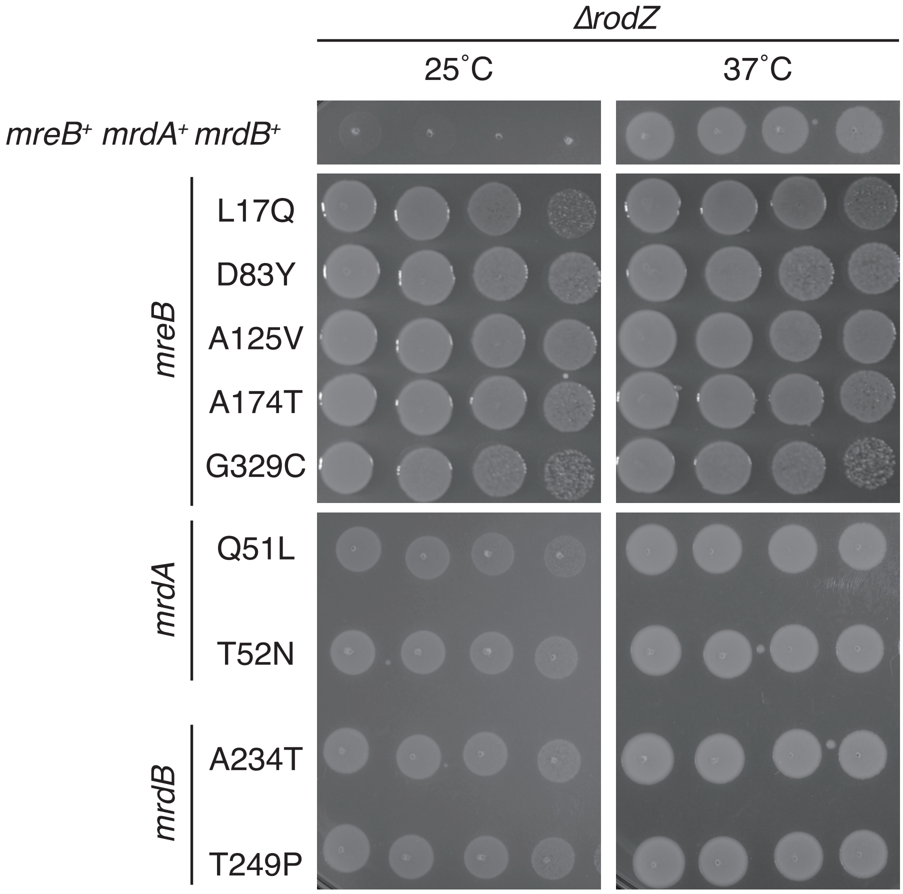

Supplementary Figure S8 Shiomi et al,

Legends to Supplementary Figures

Figure S1. Morphology of *rodZ*$^+$ or *ΔrodZ* cells producing MreB, PBP2, or RodA mutants grown in minimal medium. *rodZ*$^+$ (left) or *ΔrodZ* (right) carrying *mreB* (A) or *mrdA* or *mrdB* (B) mutation were grown in M9 medium containing 0.25% glucose at 37°C to log phase. Scale bar is 2.5 *μ*m.

Figure S2. Localization of Tar-GFP in *rodZ* mutant cells

Localization of Tar-GFP in WT, *rodZ* mutant, or *rodZ* mutant cells producing MreB-D83Y, MreB-A125V, or MreB-A174T. Cells were grown in L broth at 37°C to log phase. Scale bar is 2.5 *μ*m.

Figure S3. Alignment of MreB from *E. coli*, *C. crescentus, B. subtilis*, *T. Maritima,* and actin from *S. cerevisiae*. Suppressor mutation sites are highlighted by red boxes. Secondary structures are shown according to the three-dimensional structure of *T. Maritima* MreB (PDB, 1JCE). Helix and strand colors refer to the domain colors in Figure 4. The alignment was prepared by ClustalW.

Figure S4. Interaction between MreB and MreC. BTH101 cells producing T25-MreC fusion and T18-MreB fusion (A) or T25-MreB fusion and T18-MreC fusion (B) were streaked on P-plates containing X-gal. The plates were incubated at 30°C for 48 h.

Figure S5. Alignments of PBP2 (A) and RodA (B) from *E. coli*, *C. crescentus, B. subtilis*, and *T. maritima*. The alignments were prepared by ClustalW. (A) Red box indicates mutation sites (Q51 and T52). The first transmembrane (TM1) and dimerization domain are highlighted in gray. (B) Red boxes indicate mutation sites (A234 and T249). TM6 and TM7 are indicated in gray.

Figure S6. Models for filaments of a three-dimensional structural model of *E. coli* MreB. Three types of MreB-filaments are proposed by Salje et al., (2011): pairs of antiparallel protofilaments (A), sheets of parallel protofilaments (B), and pairs of parallel protofilaments (C). MreB filaments are shown at left, and two MreB molecules from different protofilaments are shown at right. Suppressor mutation sites are shown as in Figure 4. Membrane insertion residues are shown by cyan spheres. The cytoplasmic membrane is shown in gray.

Figure S7. Swarming abilities of *mreB* mutants. 2 *μ*l of an overnight culture of the indicated strains were spotted onto tryptone swarming plates (1% bacto tryptone, 0.5% NaCl, 0.3% agar). The plates were incubated at 33°C for 6 h (for *rodZ*$^+$) or for 12 h (for Δ*rodZ*).

Figure S8. *Ssr* mutations suppress the cold-sensitive growth of the *rodZ* mutant. An overnight culture of the indicated strains was diluted serially (from

10^{-1} to 10^{-4}) and spotted onto L plates. The plates were incubated at 25°C for 48 h or at 37°C for 24 h.

Table S1. Strains used in this study.

Strain	Description	Reference/ Source
BW25113	*rrnB ΔlacZ4787 HsdR514 Δ(araBAD)567 Δ(rhaBAD)568 rph-1*	(Datsenko & Wanner, 2000)
DS6	Same as BW25113 but Δ*rodZ::kan*	(Baba *et al.*, 2006, Shiomi et al., 2008)
DS290	Same as BW25113 but Δ*rodZ::kan*	This study
DS452	Same as BW25113 but Δ*yhdE::cat*	This study
DS454	Same as BW25113 but *ΔrodZ::kan* Δ*yhdE::cat*	This study
DS453	Same as BW25113 but Δ*yhdE::cat mreB-L17Q*	This study
DS455	Same as BW25113 but *ΔrodZ::kan* Δ*yhdE::cat mreB-L17Q*	This study
DS552	Same as BW25113 but Δ*yhdE::cat mreB-D83Y*	This study
DS629	Same as BW25113 but *ΔrodZ::kan* Δ*yhdE::cat mreB-D83Y*	This study
DS1155	Same as BW25113 but Δ*yhdE::cat mreB-D83E*	This study
DS1156	Same as BW25113 but *ΔrodZ::kan* Δ*yhdE::cat mreB-D83E*	This study
DS1159	Same as BW25113 but Δ*yhdE::cat mreB-R124C*	This study
DS1160	Same as BW25113 but *ΔrodZ::kan* Δ*yhdE::cat mreB-R124C*	This study
DS1157	Same as BW25113 but Δ*yhdE::cat mreB-R124S*	This study
DS1158	Same as BW25113 but *ΔrodZ::kan* Δ*yhdE::cat mreB-R124S*	This study

DS612	Same as BW25113 but Δ*yhdE::cat mreB-A125V*	This study
DS630	Same as BW25113 but *ΔrodZ::kan* Δ*yhdE::cat mreB-A125V*	This study
DS1165	Same as BW25113 but Δ*yhdE::cat mreB-R127H*	This study
DS1166	Same as BW25113 but *ΔrodZ::kan* Δ*yhdE::cat mreB-R127H*	This study
DS1171	Same as BW25113 but Δ*yhdE::cat mreB-E137A*	This study
DS1172	Same as BW25113 but *ΔrodZ::kan* Δ*yhdE::cat mreB-E137A*	This study
DS559	Same as BW25113 but Δ*yhdE::cat mreB-A174T*	This study
DS560	Same as BW25113 but *ΔrodZ::kan* Δ*yhdE::cat mreB-A174T*	This study
DS553	Same as BW25113 but Δ*yhdE::cat mreB-G329C*	This study
DS561	Same as BW25113 but *ΔrodZ::kan* Δ*yhdE::cat mreB-G329C*	This study
DS673	Same as BW25113 but Δ*rlpA::cat*	This study
DS674	Same as BW25113 but *ΔrodZ::kan* Δ*rlpA::cat*	This study
DS684	Same as BW25113 but Δ*rlpA::cat mrdA-Q51L*	This study
DS688	Same as BW25113 but *ΔrodZ::kan* Δ*rlpA::cat mrdA-Q51L*	This study
DS685	Same as BW25113 but Δ*rlpA::cat mrdA-T52N*	This study
DS689	Same as BW25113 but *ΔrodZ::kan* Δ*rlpA::cat mrdA-T52N*	This study
DS686	Same as BW25113 but Δ*rlpA::cat mrdB-A234T*	This study
DS690	Same as BW25113 but *ΔrodZ::kan* Δ*rlpA::cat mrdB-A234T*	This study

DS687	Same as BW25113 but Δ*rlpA::cat mrdB-AT249P*	This study
DS691	Same as BW25113 but *ΔrodZ::kan* Δ*rlpA::cat mrdB-AT249P*	This study
DS797	Same as BW25113 but Δ*rlpA*	This study
DS798	Same as BW25113 but Δ*rlpA mrdA-Q51L*	This study
DS799	Same as BW25113 but Δ*rlpA mrdB-A234T*	This study
DS1324	Same as BW25113 but Δ*rlpA mrdA-T52N*	This study
DS1325	Same as BW25113 but Δ*rlpA mrdB-T249P*	This study
KRX	For overproduction of His_6-MreB and His_6-$RodZ_{1\text{-}111}$	Promega
AH109	yeast strain for yeast two-hybrid assay	Clonthech
BTH101	Δ*cya* strain for bacterial two-hybrid assay	(Karimova *et al.*, 1998)

Table S2. Plasmids used in this study.

Plasmid	Description	Reference/ Source
pET28a	vector to express a his_6 tagged protein	Novagen
pDS725	*his_6-mreB* in pET28a [*1]	This study
pGADT7	vector to express a fusion protein with the GAL4 activation domain	Clontech
pGBKT7	vector to express a fusion protein with the GAL4 DNA-binding domain	Clontech
pDS919	*mreB* in pGADT7 [*2]	This study
pDS1020	*mreB-L17Q* in pGADT7 [*2]	This study
pDS1021	*mreB-D83Y* in pGADT7 [*2]	This study
pDS920	*mreB-A125V* in pGADT7 [*2]	This study
pDS921	*mreB-A174T* in pGADT7 [*2]	This study
pDS1022	*mreB-G329C* in pGADT7 [*2]	This study
pDS922	*mreB* in pGBKT7 [*2]	This study
pDS1023	*mreB-L17Q* in pGBKT7 [*2]	This study
pDS1024	*mreB-D83Y* in pGBKT7 [*2]	This study
pDS923	*mreB-A125V* in pGBKT7 [*2]	This study
pDS924	*mreB-A174T* in pGBKT7 [*2]	This study

pDS1025	*mreB-G329C* in pGBKT7 [*2]	This study
pT25	vector to express a fusion protein with the T25 fragment	(Karimova et al., 1998)
pKT25	vector to express a fusion protein with the T25 fragment	(Karimova et al., 1998)
pUT18C	vector to express a fusion protein with the T18 fragment	(Karimova et al., 1998)
pDS1289	*T25-mreB* in pKT25[*3]	This study
pDS1290	*T25-mreB-L17Q* in pKT25[*3]	This study
pDS1291	*T25-mreB-D83Y* in pKT25[*3]	This study
pDS1295	*T25-mreB-A125V* in pKT25[*3]	This study
pDS1298	*T25-mreB-A174T* in pKT25[*3]	This study
pDS1299	*T25-mreB-G329C* in pKT25[*3]	This study
pDS1300	*T18-mreB* in pUT18C[*3]	This study
pDS1301	*T18-mreB-L17Q* in pUT18C[*3]	This study
pDS1302	*T18-mreB-D83Y* in pUT18C[*3]	This study
pDS1306	*T18-mreB-A125V* in pUT18C[*3]	This study
pDS1309	*T18-mreB-A174T* in pUT18C[*3]	This study
pDS1310	*T18-mreB-G329C* in pUT18C[*3]	This study
pTK560	*T25-mreC* in pT25	(Kruse et al., 2005)

pTK566	*T18-mreC* in pUT18C	(Kruse et al., 2005)
pDS176	*gfp-mreB*	(Shiomi et al., 2008)
pLC113-Tar-GFP	*tar-gfp*	(Shiomi *et al*., 2006)

[*1] *mreB* was cloned as a *Sac*I-*Hind*III fragment into pET28a.

[*2] *mreB* genes were cloned as *Nde*I-*BamH*I fragments into pGADT7 or pGBKT7.

[*3] *mreB* genes were cloned as *BamH*I-*EcoR*I fragments into pKT25 or pUT18C.

References to Supplementary Tables

Baba, T., T. Ara, M. Hasegawa, Y. Takai, Y. Okumura, M. Baba, K. A. Datsenko, M. Tomita, B. L. Wanner & H. Mori, (2006) Construction of Escherichia coli K-12 in-frame, single-gene knockout mutants: the Keio collection. *Mol Syst Biol* **2**: 2006 0008.

Datsenko, K. A. & B. L. Wanner, (2000) One-step inactivation of chromosomal genes in Escherichia coli K-12 using PCR products. *Proc Natl Acad Sci U S A* **97**: 6640-6645.

Karimova, G., J. Pidoux, A. Ullmann & D. Ladant, (1998) A bacterial two-hybrid system based on a reconstituted signal transduction pathway. *Proc Natl Acad Sci U S A* **95**: 5752-5756.

Kruse, T., J. Bork-Jensen & K. Gerdes, (2005) The morphogenetic MreBCD proteins of Escherichia coli form an essential membrane-bound complex. *Mol Microbiol* **55**: 78-89.

Shiomi, D., M. Sakai & H. Niki, (2008) Determination of bacterial rod shape by a novel cytoskeletal membrane protein. *Embo J* **27**: 3081-3091.

Shiomi, D., M. Yoshimoto, M. Homma & I. Kawagishi, (2006) Helical distribution of the bacterial chemoreceptor via colocalization with the Sec protein translocation machinery. *Mol Microbiol* **60**: 894-906.
